# Supplementary material for: Tumour and host cell PD-L1 is required to mediate suppression of anti-tumour immunity in mice
Source: Nat Commun. 2017 Feb 21;8:14572. doi: 10.1038/ncomms14572 (PMC5321797; doi:10.1038/ncomms14572)
Supplement: Supplementary Information — Supplementary Figures and Supplementary Table [file ncomms14572-s1.pdf]

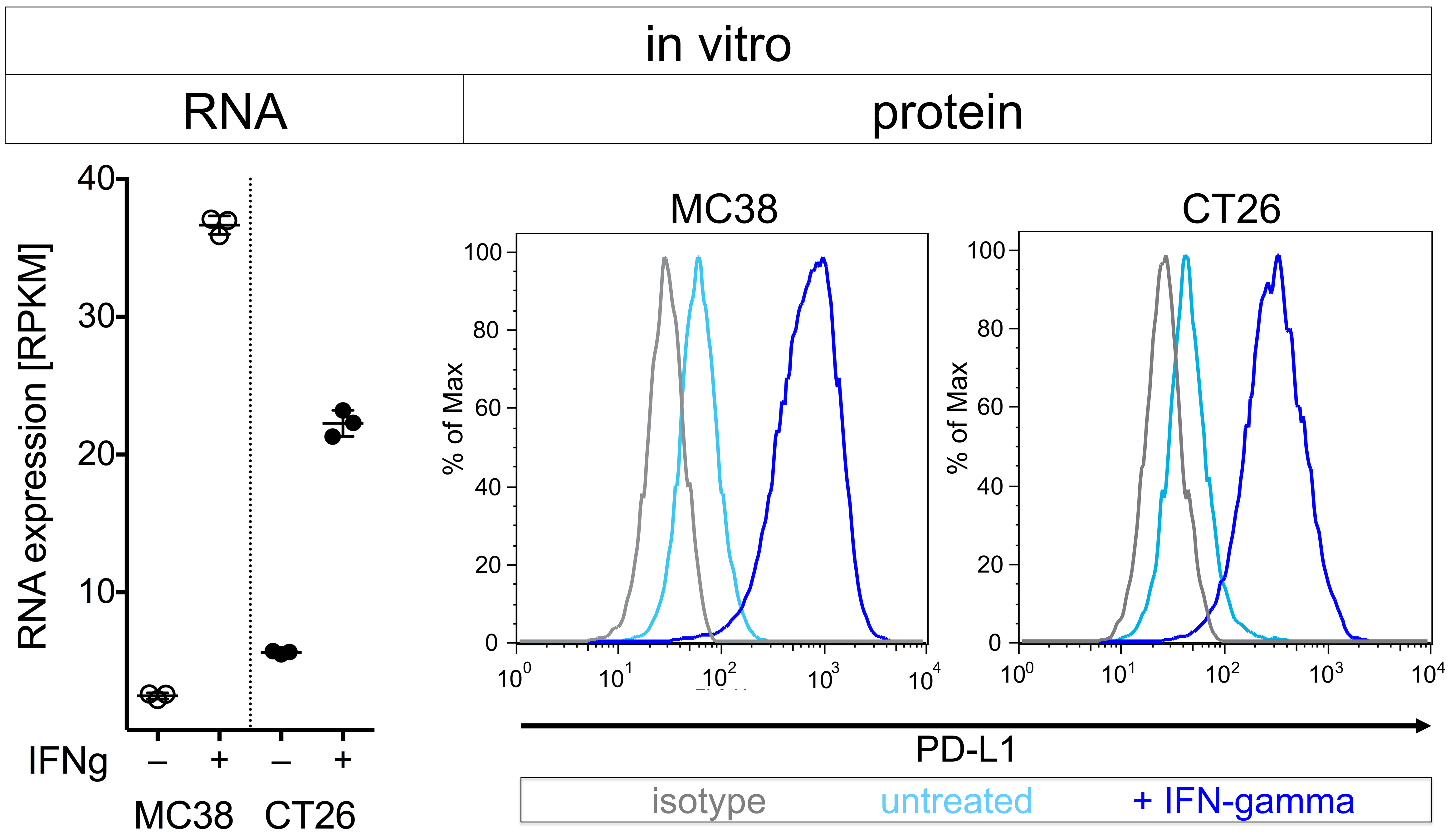

Supplementary Figure 1 In vitro PD-L1 expression in MC-38 and CT-26 tumour cells can be induced by IFNg exposure

In vitro RNA and surface protein expression of PD-L1 in MC-38 or CT-26 tumour cells can be induced by IFNγ. Data are representative of three independent studies with three technical replicates per treatment group. Error bars depict SD from the mean.

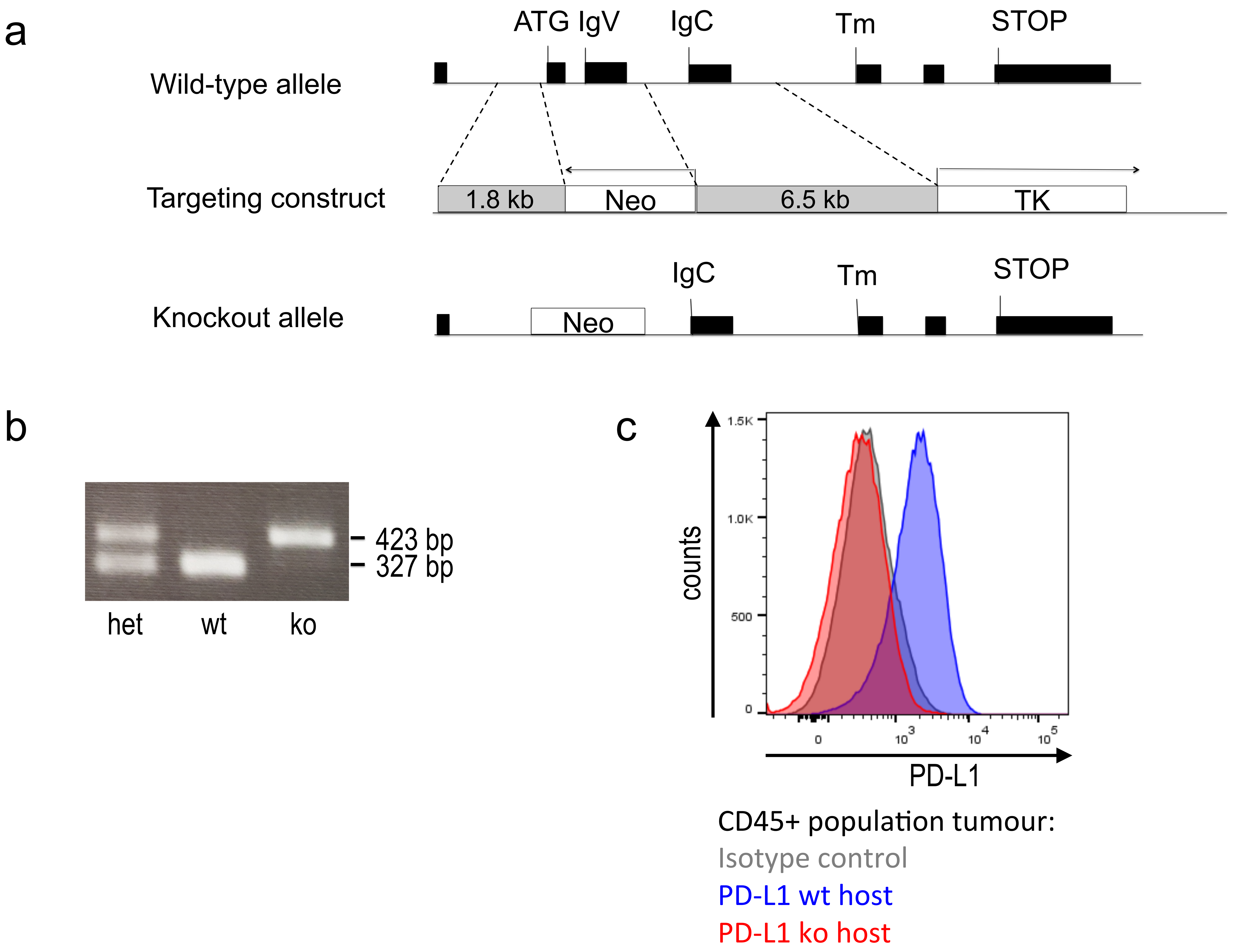

Supplementary Figure 2 ***mPd-l1* targeting strategy to generate PD-L1 deficient host mice**

Schematic of mouse *pd-l1/cd274* locus showing targeting construct for knockout allele (a). Genotyping by PCR confirmed allelic loss in off-springs from F2^{+} het x het crosses: wild-type (wt), heterozygous (het) or PD-L1 deficient knockout (ko) mice (b). Flow cytometry analysis confirmed loss of PD-L1 surface expression in CD45+ cells isolated from MC38 tumours implanted into PD-L1 wt or ko host mice (c). Isotype control=grey; PD-L1 host wt = blue; PD-L1 host ko=red. Neo: Neomycin cassette; TK: thymidine kinase

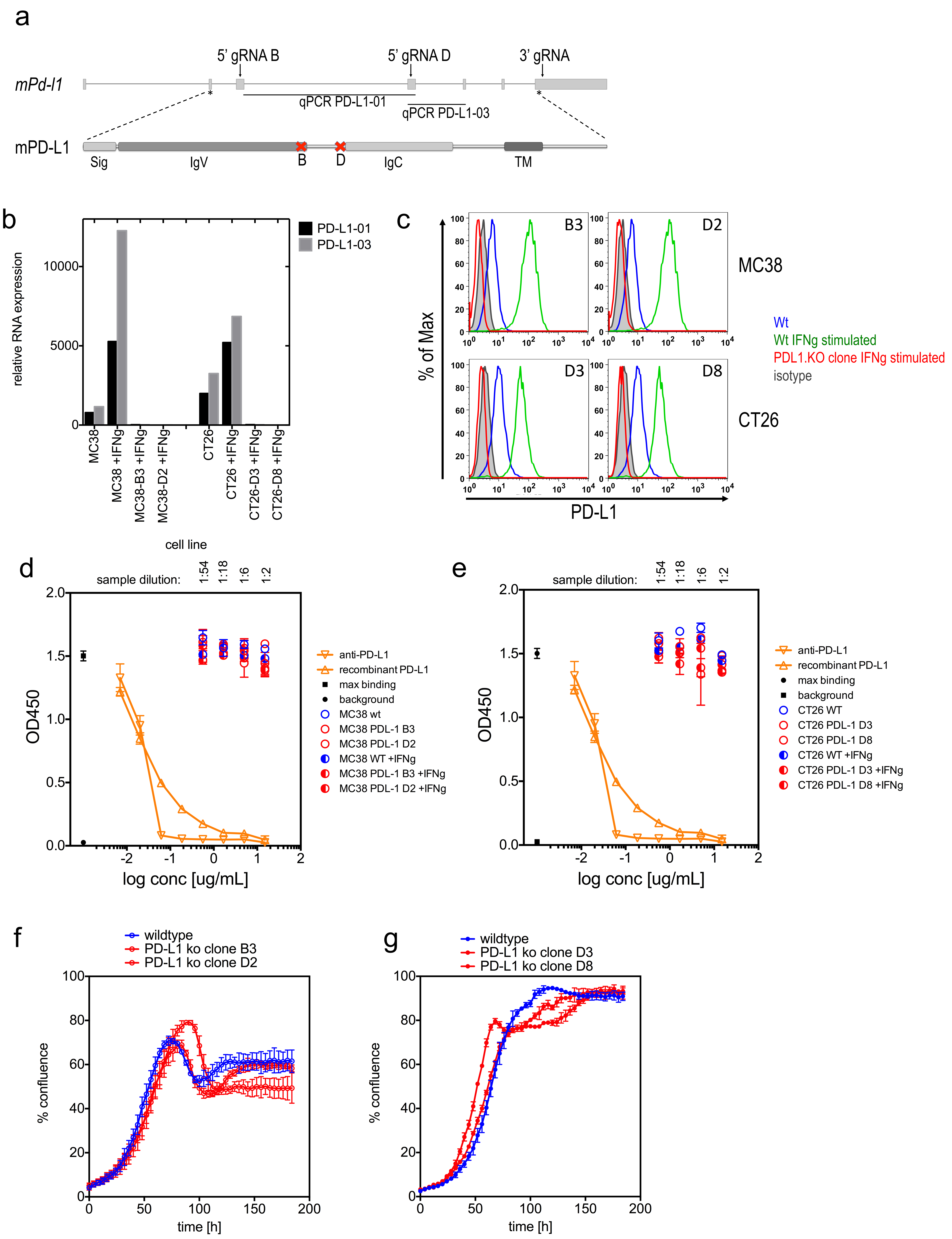

a
5' gRNA B
5' gRNA D
3' gRNA
mPd-l1
qPCR PD-L1-01
qPCR PD-L1-03
mPD-L1
Sig
IgV
B
D
IgC
TM
b
relative RNA expression
PD-L1-01
PD-L1-03
MC38
MC38 +IFNg
MC38-B3 +IFNg
MC38-D2 +IFNg
CT26
CT26 +IFNg
CT26-D3 +IFNg
CT26-D8 +IFNg
cell line
c
% of Max
B3
D2
D3
D8
MC38
CT26
Wt
Wt IFNg stimulated
PDL1.KO clone IFNg stimulated
isotype
PD-L1
d
sample dilution:
1:54
1:18
1:6
1:2
OD450
log conc [ug/mL]
anti-PD-L1
recombinant PD-L1
max binding
background
MC38 wt
MC38 PDL-1 B3
MC38 PDL-1 D2
MC38 WT +IFNg
MC38 PDL-1 B3 +IFNg
MC38 PDL-1 D2 +IFNg
e
sample dilution:
1:54
1:18
1:6
1:2
OD450
log conc [ug/mL]
anti-PD-L1
recombinant PD-L1
max binding
background
CT26 WT
CT26 PDL-1 D3
CT26 PDL-1 D8
CT26 WT +IFNg
CT26 PDL-1 D3 +IFNg
CT26 PDL-1 D8 +IFNg
f
wildtype
PD-L1 ko clone B3
PD-L1 ko clone D2
% confluence
time [h]
g
wildtype
PD-L1 ko clone D3
PD-L1 ko clone D8
% confluence
time [h]

Supplementary Figure 3 Genetic deletion of PD-L1 in CT26 and MC38 colon cancer lines utilizing the CAS9/CRISPR system

Schematic of mouse *pd-l1/cd274* locus showing location of 5' and 3' gRNAs for targeted gene deletion by CAS9/CRISPR, qPCR primer/probe set location (qPCR PD-L1 01 and 03) and putative protein truncation resulting from deletion of exon 3-7 (B clones) or exon 4-7 (D clones) (a). PD-L1 deficient single cell clones were generated, and PD-L1 loss confirmed by qPCR (b) and flow cytometry (c). Supernatants of control and IFNγ stimulated wild-type and PD-L1 deficient MC38 (d) and CT26 (e) clones were tested for presence of any putative truncated protein that might interfere with PD-1/PD-L1 interaction, but no such activity was detected. PD-L1 deficient MC38 (f) and CT26 (g) clones show normal in vitro proliferation. Experiments show representative data from three technical replicates. Error bars depict SD from the mean.

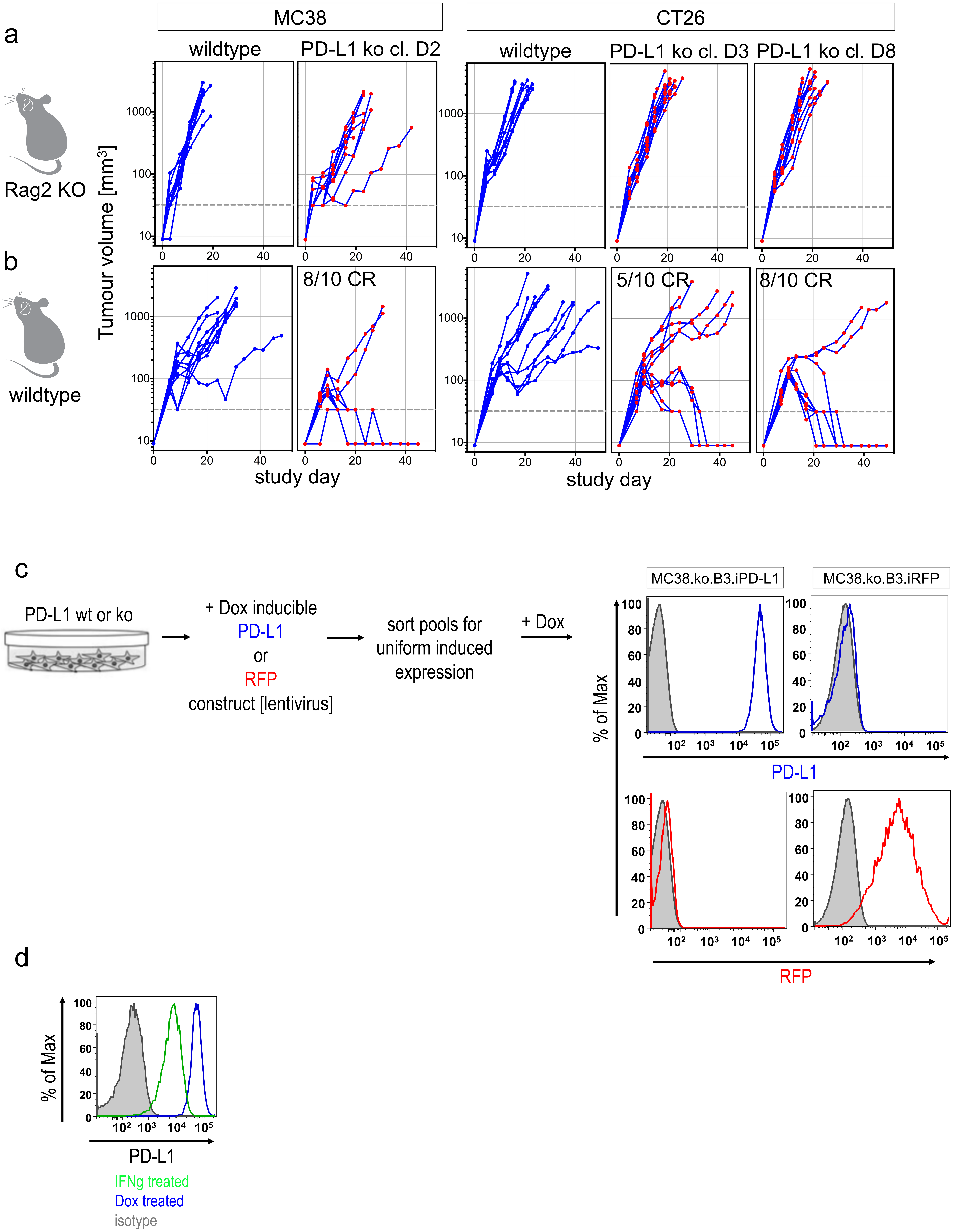

a
b
c
d
MC38
CT26
wildtype
PD-L1 ko cl. D2
PD-L1 ko cl. D3
PD-L1 ko cl. D8
Rag2 KO
wildtype
Tumour volume [mm3]
8/10 CR
5/10 CR
8/10 CR
study day
PD-L1 wt or ko
+ Dox inducible
PD-L1
or
RFP
construct [lentivirus]
sort pools for
uniform induced
expression
+ Dox
MC38.ko.B3.iPD-L1
MC38.ko.B3.iRFP
% of Max
PD-L1
RFP
IFNg treated
Dox treated
isotype

Supplementary Figure 4 Loss of PD-L1 in MC38 and CT26 colon cancer cells leads to tumour rejections in immune competent hosts

Tumour growth kinetics and frequency of complete regressions (CR) for additional PD-L1 deficient MC38 and CT26 clones were tested in immune deficient Rag2-/- (a) and immune competent recipient mice (b). Data from PD-L1 wild-type (wt) tumours is represented in blue and PD-L1 deficient (ko) tumours represented in red, from two independent studies with ten animals/group. Wild-type and PD-L1 deficient MC-38 and CT-26 cells were transduced with a in a pInducer11 lentiviral vector encoding constitutive GFP and doxycycline-inducible PD-L1 cDNA (iPD-L1) or RFP, and pools sorted for uniformly induced PD-L1 or RFP expression (c). Flow cytometry of PD-L1 surface levels in doxycycline inducible lines relative to endogenous levels induced by IFNγ (d). Mouse cartoon adapted from [15].

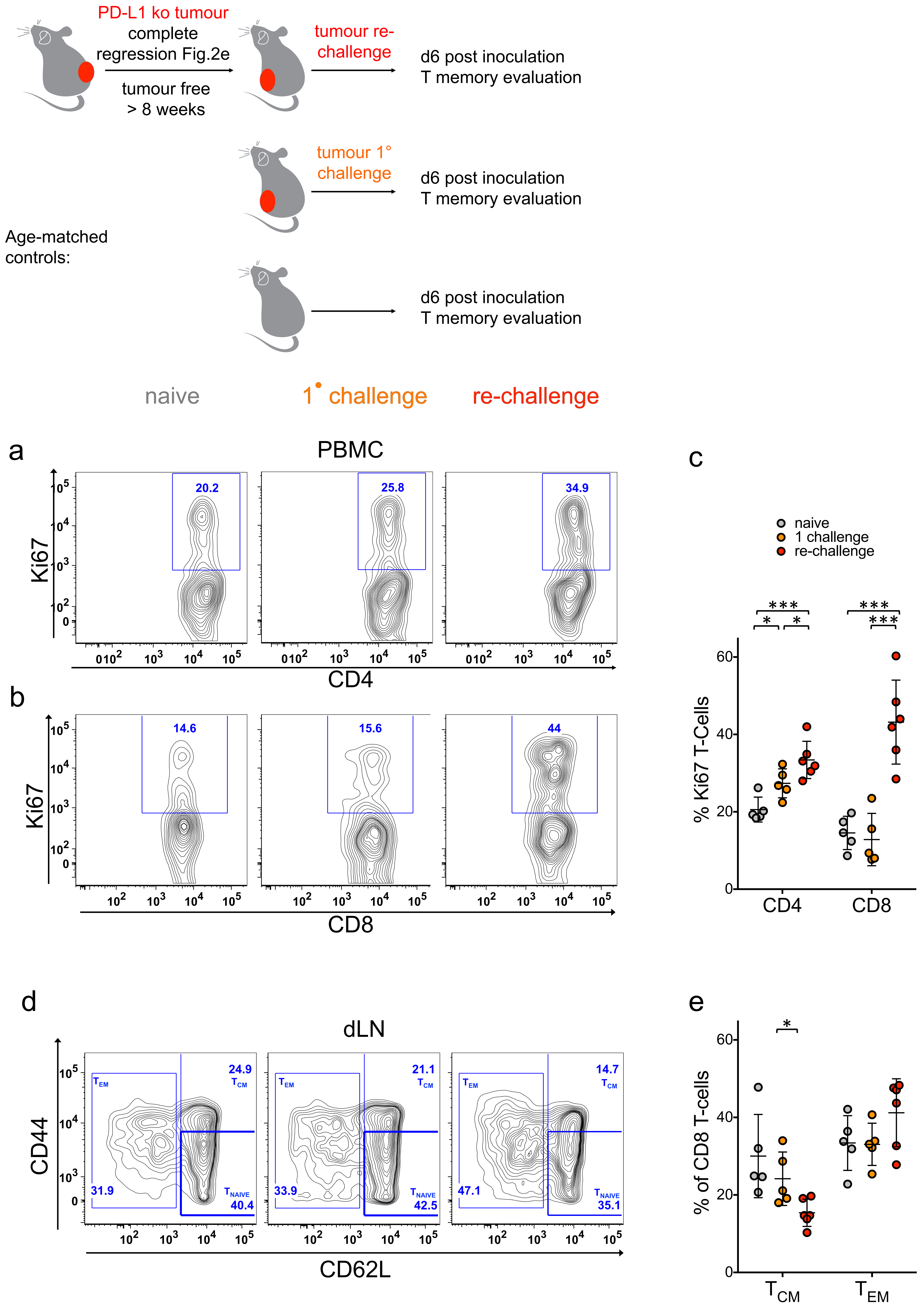

PD-L1 ko tumour
complete
regression Fig.2e
tumour free
> 8 weeks
tumour re-
challenge
d6 post inoculation
T memory evaluation
tumour 1°
challenge
d6 post inoculation
T memory evaluation
Age-matched
controls:
d6 post inoculation
T memory evaluation
naive
1° challenge
re-challenge
a
PBMC
Ki67
CD4
20.2
25.8
34.9
b
Ki67
CD8
14.6
15.6
44
c
naive
1 challenge
re-challenge
% Ki67 T-Cells
CD4
CD8
d
dLN
CD44
CD62L
T_EM
T_CM
T_NAIVE
24.9
31.9
40.4
21.1
33.9
42.5
14.7
47.1
35.1
e
% of CD8 T-cells
T_CM
T_EM

Supplementary Figure 5 Immune reactivity in PD-L1 deficient tumors leads to productive memory formation

Following complete regression of PD-L1 deficient CT26 tumours, mice show increased CD4+ (a,c) and CD8+ (b,c) proliferation in the periphery six days after re-challenge with the same tumour cell line. CD8+ T-cells in draining lymph nodes shift from central memory (CD62L+, CD44+) to effector memory (CD62L-CD44+) cells when compared to controls (d, e). Data shown for age-matched naïve mice (grey circles), and mice receiving primary inoculation (orange circles) or mice re-challenged (red circles) with PD-L1 deficient CT26 cells at day 6. Data is representative of five technical replicates from two independent studies. Statistical significance was determined by Student t-test (p value *<0.05; **<0.01; ***<0.001.) PBMC: peripheral blood mononuclear cells; dLN: tumour draining lymph node. Error bars depict SD from the mean. Mouse cartoon modified from [15].

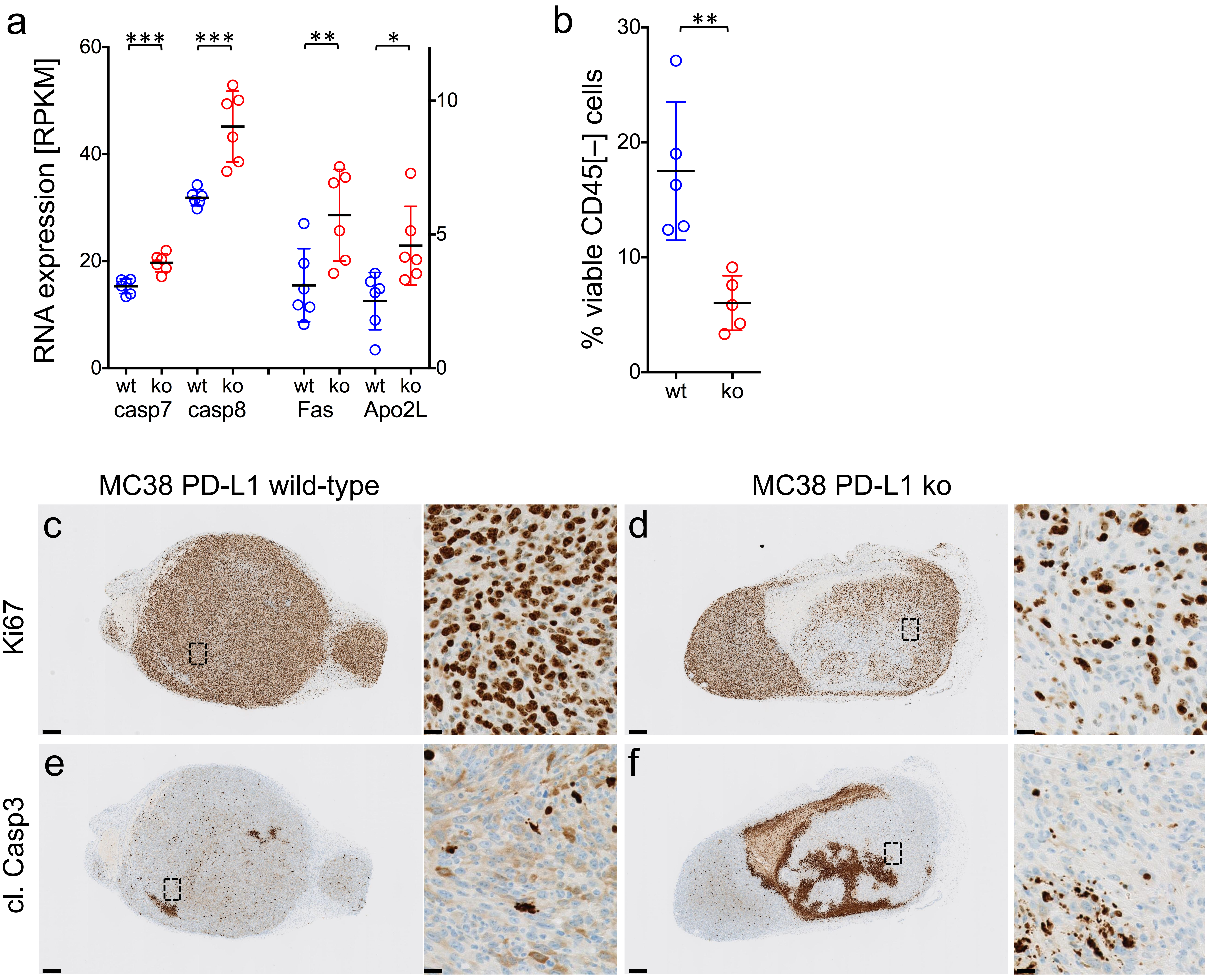

Supplementary Figure 6 Outgrowing PD-L1 deficient tumors are characterized by increased cell death and reduced cell proliferation

RNASeq analysis of PD-L1 deficient outgrowing tumours harvested at 250 mm^3 show an increase in apoptotic gene expression compared to size matched wild-type (wt) tumours harvested at the same time point (a), consistent with a reduction of tumour cell viability as determined by flow cytometry in the CD45- population (b). Established wild-type (c, e) and PD-L1 deficient (d, f) tumours (at 250mm^3) evaluated for Ki67 (c, d), and cleaved caspase 3 (e, f) by IHC confirmed reduced proliferation and increased cell death in tumours, though caspase 3 levels were similar to wt tumours outside the large necrotic areas (insert). Scale bars: 250 µm in wide-field images, and 20 µm in high magnification image indicated by hatched boxes. Data is representative of five technical replicates from two independent study repeats. Statistical significance was determined by Student t-test (p value *<0.05; **<0.01; ***<0.001) except for RNASeq data which is reported as q-value to adjust for multiple testing (q value *<0.1; **<0.01; ***<0.001). Error bars depict SD from the mean.

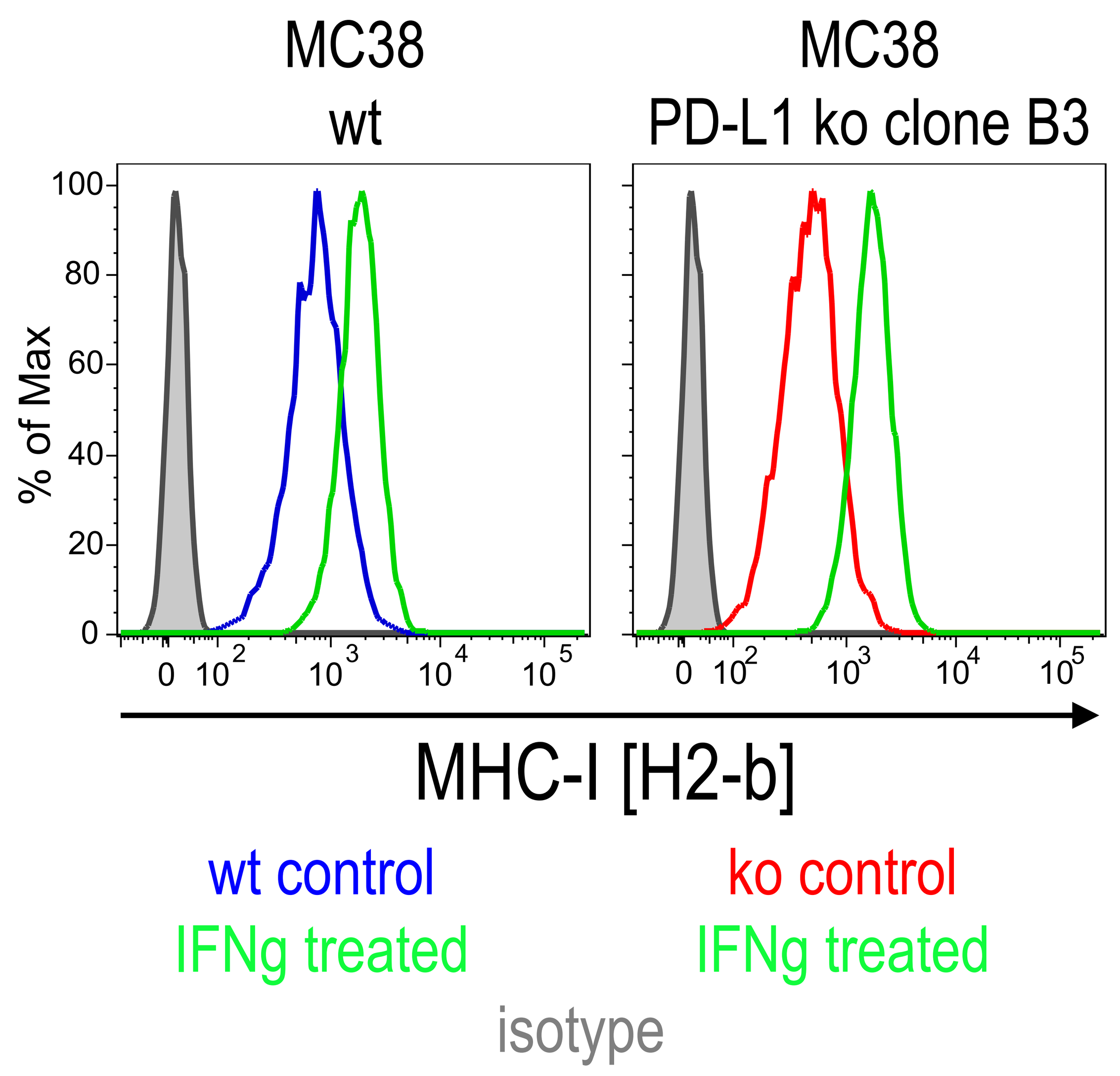

Supplementary Figure 7 PD-L1 deficient clones show normal MHC I expression in vitro

MC38 wild-type (wt, blue) and PD-L1 deficient (ko, red) cells were assessed for MHC I surface levels by flow cytometry. Baseline and IFNg induced (green) surface levels were comparable for wt and ko clones. Isotype: grey.

Supplementary table 1 Primer and probe sets used for qRT-PCR gene expression analysis normalization

Gene	Forward Primer	Reverse Primer	Probe (FAM/BHQ1)
ACTB	TCTGAATGGCCCAGGTCT	CTGCCTCAACACCTCAACC	CCCTCCCAGGGAGACCAAAGC
RPS13	CACCGATTGGCTCGATACTA	TAGAGCAGAGGCTGTGGATG	CGGGTGCTCCCACCTAATTGGA
HMBS	CTCAGTGTCCTGTTGCTGCT	TTCTTCCTTCTGCCCTCCTA	TGGTCTTGCTTCTCTGGATCCCG
